# The safety of esophageal cancer surgery during COVID-19

**DOI:** 10.1097/MD.0000000000030929

**Published:** 2022-10-14

**Authors:** Qiuxiang Wang, Chengjiao Yao, Yilin Li, Lihong Luo, Fengjiao Xie, Qin Xiong, Ruike Wu, Juan Wang, Peimin Feng

**Affiliations:** a Chengdu University of Traditional Chinese Medicine Affiliated Hospital, Chengdu, Sichuan Province, China; b Department of Traditional Chinese Medicine, The Central Hospital of Guangyuan City, Sichuan Province, China; c Department of Geriatrics of the Affiliated Hospital, Nanchong, Sichuan Province, China.

**Keywords:** COVID-19 pandemic, esophageal cancer, meta-analysis, protocol, surgery, systematic review

## Abstract

The ongoing coronavirus disease 2019 (COVID-19) pandemic has drastically challenged the safety of on esophageal cancer (EC) surgery during COVID-19. The study aimed to evaluate the safety of EC surgery during the COVID-19 pandemic.

**Methods:**

This systematic review was performed in accordance with the PRISMA-P 2015 guidelines and registered in PROSPERO (registration number: CRD42022335164). A systematic search of PubMed, Embase, Cochrane Library, Web of Science, Medline, Chinese National Knowledge Infrastructure database, Chinese Scientific Journal database, and Wan Fang database was conducted to identify potentially relevant publications from January 2020 to May 2022. All data were independently extracted by two researchers. We will apply a fixed-effect model or random effect model basis on the heterogeneity test and employ with RevMan 5.4.1 software for data synthesis. The dichotomous surgical outcomes used risk ratios or risk differences, and for continuous surgical outcomes, mean differences (MD) or standardized MD, both with 95% confidence intervals were used. The primary outcomes were postoperative complications, anastomotic leaks, and mortality. The secondary outcomes were total hospital stay, postoperative stay, preoperative waiting, operation time, blood loss, transfusion, postoperative intensive care unit (ICU) stay, number of patients needing ICU stay, and 30-day readmission.

**Results:**

This study will comprehensively summarize the high-quality trials to determine the safety of EC surgery during COVID-19.

**Conclusion:**

Our systematic review and meta-analysis will present evidence for the safety of EC surgery during COVID-19.

## 1. Introduction

The ongoing coronavirus disease 2019 (COVID-19) pandemic caused by the severe acute respiratory syndrome coronavirus 2 (SARS-CoV-2) has posed a serious public health threat worldwide, with millions of people at risk in a growing number of countries.^[1]^Up to May 22, 2022, over 522 million confirmed cases and over 6 million confirmed deaths have been reported to the World Health Organization from different countries, areas and territories. During the pandemic, the management of patients with cancer has been affected at multiple stages, including the triage decisions, surgery, and neoadjuvant therapy as a bridge to reduce admissions and preserve health-care resources.^[2]^ COVID Surg Collaborative estimated that 28,404,603 elective operations were canceled or postponed worldwide during the 12 weeks of peak disruption, with 38% being for cancer.^[3]^

Esophageal cancer (EC) is the 8th most common type of cancer worldwide, constitutes the sixth leading cause of cancer deaths.^[4]^ EC remains a global health concern with a dismal prognosis and an estimated 5-year survival rate of approximately 10% to 15%.^[5]^ Surgery plays an important role in the treatment strategies for EC. Recent advances in surgical techniques and perioperative management have dramatically improved the mortality rate; however, esophagectomy remains a highly invasive procedure that can lead to severe postoperative complications.^[6]^ Besides, the rapid global spread of COVID-19 presents an unprecedented crisis for esophagectomy.^[7]^ Recent reports suggest that patients with cancer might have a higher risk of COVID-19 than individuals without cancer and patients with cancer had poorer outcomes from COVID-19.^[8]^ In particular, for patients with EC, additional precautions have been taken, as surgery for EC alone has higher morbidity and mortality rates compared to that for other oncological surgeries.^[9]^ The complexity of the multidisciplinary approach to EC patients and the high morbidity rates of esophageal surgery have challenged the treatment pathways of these patients, especially during the COVID-19 pandemic. Overall, 55% of scheduled endoscopic resections for gastrointestinal neoplastic lesions were deferred globally after the lockdown period, which was 11 times higher than in the previous year, and the majority of postponements (80%) occurred in severely affected countries.^[10]^ For patients with cancer, delay of surgery has the potential to increase the likelihood of metastatic disease, with some patients’ tumors progressing from being curable (with near-normal life expectancy) to noncurable (with limited life expectancy).^[11]^ Delay in time to esophagectomy for EC has been shown to have worse perioperative and long-term outcomes. Therefore, general guidance from health ministries and national surgical associations supported that time-dependent surgery should continue.^[12]^ Despite outbreaks, cancer surgery must continue to prevent an overwhelming number of delayed operations, a possible increase in emergency procedures, and a significant decline in population health.^[3]^

However, there have been conflicting results regarding the safety of EC surgery during the COVID-19 pandemic. Some studies have demonstrated that EC surgical procedures may be safely performed during the pandemic.^[13–17]^ while another study reported that one of the esophago-gastric junction cancer patients developed COVID-19 pneumonia on post-operative day two, leading to impaired respiratory function and increased pleural fluid collection from the chest tube, resulting in a prolonged hospital stay.^[18]^ And the other study reported the case of a young man who underwent thoracoscopic subtotal esophagectomy for distal esophageal adenocarcinoma who developed COVID-19 with severe clinical presentation.^[19]^ Modeling the impact of delaying surgery for early EC in the era of COVID-19 showed that as the risk of infection with COVID-19 increased above 7%, delaying operations for 3 months has an improved long-term survival.^[20]^ While other study showed that the EC time to surgery over 8 weeks is associated with lower survival.^[21]^ So it is necessary to evaluate the safety of EC surgery during COVID-19. To the best of our knowledge, there have been no meta-analyses on the safety of EC surgery during COVID-19. Therefore, we performed a meta-analysis of cohort studies to evaluate the safety of EC surgery during the COVID-19 pandemic.

## 2. Methods

### 2.1. Study registration

The present study was conducted in accordance with the preferred reporting items for systematic reviews and meta-analysis protocols statement guidelines^[22]^ and has been registered with PROSPERO under registration number CRD42022335164.

### 2.2. Selection criteria

#### 2.2.1. Type of studies.

The present study is a cohort studies of the safety of EC surgery during COVID-19.

#### 2.2.2. Types of participants.

The sample population included patients diagnosed with EC. The subjects enrolled were EC patients undergoing surgery before COVID-19 and during COVID-19, and there were no restrictions on the type of surgery.

#### 2.2.3. Types of interventions and comparisons.

However, the intervention is not applicable. This meta-analysis will evaluate the safety of colorectal cancer surgery during COVID-19. Postoperative complications, anastomotic leak, mortality, total hospital stay, postoperative stay, preoperative waiting time, operation time, blood loss, transfusion, postoperative intensive care unit (ICU) stay, the number of patients needing ICU stay, and 30-day readmission were compared to evaluate the safety of EC surgery before and during COVID-19.

#### 2.2.4. Language.

There is restriction on Chinese or English.

### 2.3. Exclusion criteria

Studies that met the following criteria were excluded: duplicate studies; articles published as case reports, case series, review articles, letters, editorials, and commentaries will be excluded; studies involving data that cannot be extracted or inadequate are lacking; there is no control group that assesses the safety of EC surgery during the pre-COVID-19 period; no outcome measures of interest are reported; Newcastle-Ottawa Scale (NOS) scores <5 points; studies written in languages other than Chinese or English.

### 2.4. Types of outcome measures

#### 2.4.1. Primary outcomes.

The primary outcomes of this study were to evaluate the postoperative complications, mortality, and anastomotic leak after EC surgery before COVID-19 and during COVID-19.

#### 2.4.2. Secondary outcomes.

The secondary outcomes of this study were to evaluate the total hospital stay, postoperative stay, preoperative waiting time, operation time, blood loss, transfusion, postoperative ICU stay, the number of patients needing ICU stay, and 30-day readmission of EC surgery before COVID-19 and during COVID-19.

### 2.5. Search strategy

A systematic search plan will be performed in the following eight databases with a time restriction from January 2020 to May 2022 to filter eligible studies: PubMed, Embase, the Cochrane Library, Web of Science, Medline, Chinese National Knowledge Infrastructure database, Chinese Scientific Journal database, and Wan Fang database. We also will search journal articles, conference papers, and academic papers. The search keywords included terms related to COVID-19 pandemic, terms related to EC and terms related to surgery. Cross references were checked to assess if any relevant studies were missed. We considered a specific search strategy in PubMed as a typical example; the specific steps of the retrieval are shown in Table 1.

**Table 1:** Search strategy in PubMed database.

Search items
#1 Esophageal Neoplasms [Mesh]
# 2 Neoplasm, Esophageal [Title/Abstract]
#3 Esophagus Neoplasm [Title/Abstract]
#4 Neoplasm, Esophagus [Title/Abstract]
#5 Cancer of Esophagus [Title/Abstract]
#6 Cancer of the Esophagus [Title/Abstract]
#7 Esophagus Cancer [Title/Abstract]
#8 Cancer, Esophagus [Title/Abstract]
#9 Cancers, Esophagus [Title/Abstract]
#10 Esophagus Cancers [Title/Abstract]
#11 Esophageal Cancer [Title/Abstract]
#12 Cancer, Esophageal [Title/Abstract]
#13 Cancers, Esophageal [Title/Abstract]
#14 Esophageal Cancers [Title/Abstract]
#15 or #1–#14
#16 surgery [Title/Abstract]
#17 operative therapy [Title/Abstract]
#18 operative procedures [Title/Abstract]
#19 operations [Title/Abstract]
#20 perioperative procedures [Title/Abstract]
#21 intraoperative procedures [Title/Abstract]
#22 or #16–#21
#23 COVID-19 Pandemic [Title/Abstract]
#24 COVID 19 Pandemic [Title/Abstract]
#25 Pandemic, COVID-19 [Title/Abstract]
#26 COVID-19 Pandemics [Title/Abstract]
#27 or #23–#26
#28 #15 and #22 and #27

### 2.6. Data collection and analysis

#### 2.6.1. Study selection.

**Figure 1.**
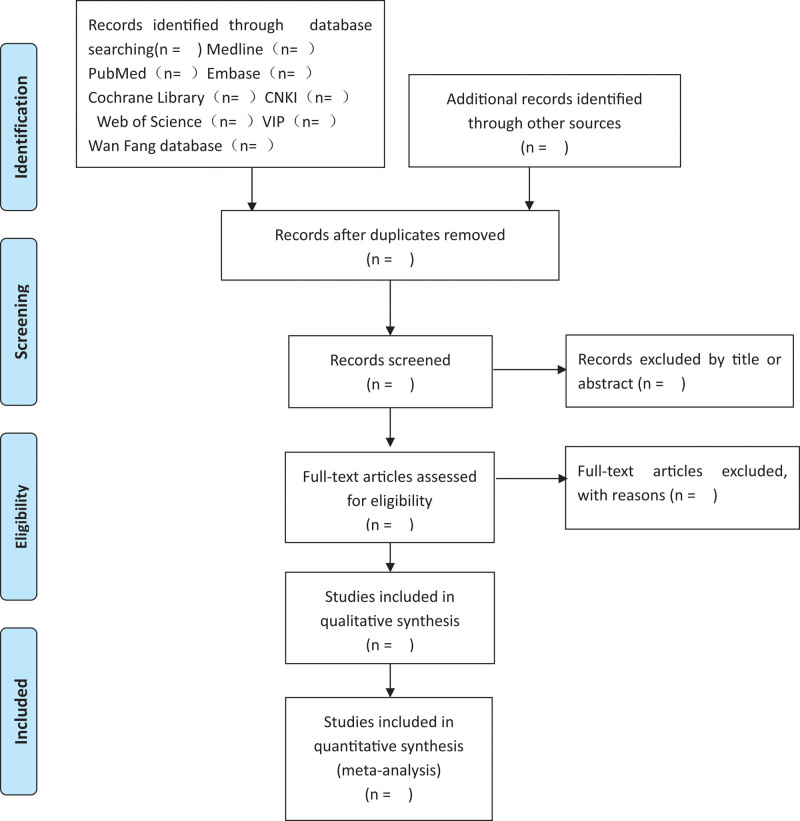
The guideline flow diagram.

We will rely on two independent authors (QW and RW) to screen and select the eligible studies. The literature obtained will be imported into NoteExpress to screen the title and abstract. Subsequently, we will obtain full-text articles from relevant studies. After reading the full text of the remaining studies, the final number of included studies will be determined. Disagreements will be resolved by a third reviewer (PF). The entire study selection process is presented in the guideline flow diagram (Fig. 1).

#### 2.6.2. Data extraction.

Two reviewers (QW and JW) will independently extract data from the included studies and enter the extracted data into Excel sheets. Disagreements will be resolved through negotiation and discussion. Further controversy will be arbitrated by a third reviewer (PF). The following information was extracted from each included study: study baseline (the first author’s name, published year, country, study design, study size, age, sex, operative methods); primary surgical outcomes: postoperative complications, anastomotic leak, mortality; secondary surgical outcomes: total hospital stay, postoperative stay, preoperative waiting, operation time, blood loss, transfusion, postoperative ICU stay, the number of patients needing ICU and 30-day readmission.

#### 2.6.3. Assessment of risk of bias.

NOS was used for the quality assessment of the included studies.^[23]^ NOS provides a checklist of items for evaluating the quality of reporting and the risk of bias of the included studies based on three broad evaluation categories: selection, comparability, and exposure/outcomes.^[24]^ The scale has three parameters and eight items with a total score of 9, scores ≤ 3 are usually considered low quality, scores of 4 or 5 are considered medium quality, and scores ≥ 6 are usually considered high quality.^[25]^ Two reviewers (FX and RW) independently performed the quality assessment of the included studies, and the third team member (PF) performed the verification. Discrepancies were resolved through discussion.

#### 2.6.4. Measures of perioperative outcomes.

For dichotomous data, such as postoperative complications, anastomotic leak, mortality, number of patients requiring ICU admission, and 30-day readmission, we plan to present the results as risk ratios (RR) with 95% confidence intervals (CIs). For continuous data, such as total hospital stay, postoperative stay, preoperative waiting, operation time, blood loss, transfusion, and postoperative ICU stay, we will use the MD with a 95% CIs. Statistical significance was set at *P* < .05.

#### 2.6.5. Data analysis and heterogeneity processing.

We will use the Review Manager (RevMan) software (Version 5.4, Copenhagen: The Nordic Cochrane Centre, The Cochrane Collaboation,s 2020) for the meta-analysis and statistical analysis. Egger’s test was performed using the Stata software (version 16.0, Stata Corp LP, College Station). Heterogeneity among the studies was evaluated using chi-square tests and inconsistency statistic.^[26]^ If the included article reported outcomes in medians and interquartile ranges, the method described by Wan et al was used to calculate the mean and standard deviation (SD).^[27,28]^ If the included articles reported outcomes as medians, maximum and minimum, the method described by Hozo et al was used to calculate the mean and SD.^[29]^It indicated that there was significant heterogeneity if *I*^2^ > 50% and/or *P* < .1, the fixed-effects model (Mantel–Haensze) was used to analyze the data if no heterogeneity was present, whereas the random-effects model was used if I^2^ > 50%.^[30]^ The levels of heterogeneity assessed using *I*^2^ were as follows: 0% to 25%, homogeneity; 25% to 50%, low heterogeneity; 50% to 75%,moderate heterogeneity; and > 75% meant high heterogeneity.^[31]^ Possible reasons for heterogeneity will be determined using sensitivity analysis or subgroup analysis. A descriptive analysis of the results will be performed when considerable heterogeneity makes the analysis infeasible.

#### 2.6.6. Subgroup analysis.

Subgroup analyses were conducted based on different type of surgical methods.

#### 2.6.7. Sensitivity analysis.

The stability of the meta-analysis results was tested by changing effect model method in sensitivity analysis.

#### 2.6.8. Assessment of reporting bias.

When we select > 10 studies consistent with these conditions, a funnel plot was used to detect publication bias, and the Egger test of bias was used as a supplement.^[32]^

#### 2.6.9. Ethics and dissemination.

This systematic review and meta-analysis was based on published data. As the researchers did not access any information that could lead to the identification of an individual patient, no ethical issues were raised in this study. Therefore, the requirement for ethical approval and consent from participants was waived. The research results may be published in peer-reviewed journals or disseminated at relevant conferences.

#### 2.6.10. Grading the quality of evidence.

The Grading of Recommendations Assessment, Development and Evaluation guidelines will be used to grade the quality of evidence as very low, low, moderate, or high, respectively.

## 3. Discussion

As COVID-19 prevention and control have become normal, the situation of COVID-19 is still not optimistic. During the COVID-19 pandemic, patients with EC are at a higher risk of developing COVID-19 due to frequent hospital visits and an increased risk of developing severe disease after the infecting COVID-19. Surgery is the main treatment modality for solid cancers.^[33]^ Therefore, whether EC surgery can be performed safely during the COVID-19 pandemic has become an important topic of clinical concern.

Esophagectomy is a complex surgical procedure and is associated with substantial morbidity, particularly postoperative pneumonia and consecutive respiratory failure.^[34–37]^ An added risk of COVID-19 may be on increasing the risk of anastomotic leaks, as has been reported for colorectal cancer resections, as anastomotic leaks are reported in between 5% and 25% of EC cases and may have fatal consequences.^[34,35,38]^ Moreover, patients with EC have poor nutrition and low resistance, which are associated with a high risk of anastomotic leaks. The shortage of ICU facilities and the potential increased risk of mortality related to perioperative COVID-19 infection in cancer patients have raised several concerns about the most appropriate surgical management of patients with EC.^[15]^ So it is necessary to evaluate the safety of EC surgery during the COVID-19 pandemic.

However, no systematic review or meta-analysis has been conducted on the safety of EC surgery during COVID-19. This study systematically evaluated the safety of EC surgery during COVID-19, in order to provide evidence-based evidence for the safety EC surgery during COVID-19. In this systematic review and meta-analysis, we will investigate clinical studies on the safety of EC surgery during COVID-19 by assessing the perioperative results, including: postoperative complications, anastomotic leak and mortality, total hospital stay, postoperative stay, preoperative waiting time, operation time, blood loss, transfusion, postoperative ICU stay, number of patients needing ICU, and 30-day readmission. We expect that our findings will be a useful resource for clinical practitioners and patients to suggest optimized clinical surgical strategies for EC during COVID-19.

## Author contributions

**Conceptualization:** Qiuxiang Wang.

**Data curation:** Qiuxiang Wang, Chengjiao Yao, Yilin Li, Ruike Wu.

**Formal analysis:** Qiuxiang Wang, Chengjiao Yao.

**Funding acquisition:** Lihong Luo, Qin Xiong.

**Investigation:** Qiuxiang Wang, Yilin Li.

**Methodology**: Qiuxiang Wang, Chengjiao Yao, Fengjiao Xie.

**Supervision:** Peimin Feng.

**Writing – original draft:** Qiuxiang Wang.

**Writing – review & editing:** Qiuxiang Wang, Chengjiao Yao, Juan Wang.
